# CellScore: an open-source image-based pipeline for automated cell quantification in microalgae

**DOI:** 10.1007/s44307-026-00131-w

**Published:** 2026-11-09

**Authors:** Sally Do, Shijie Huang, Angelyn Plan, Noah Birn, Alexander Walker, Zhi-Yan Du

**Affiliations:** 1 Department of Molecular Biosciences & Bioengineering, University of Hawai’i at Mānoa, Honolulu, HI 96822 USA; 2 Department of Ocean and Resources Engineering, University of Hawai’i at Mānoa, Honolulu, HI 96822 USA

**Keywords:** Automated cell counting, Open-source software, Microscopy

## Abstract

Accurate quantification of microalgal cell density is essential for both basic research and industrial applications, as it directly reflects culture growth, productivity, and physiological status. There are existing methods routinely used to measure cell counts, although many are limited by high cost, labor-intensive workflows, or steep technical barriers. To address these limitations, we developed CellScore, a rapid and accessible image-based pipeline for microalgal cell counting implemented in ImageJ, a widely adopted open-source platform in the biological sciences (https://github.com/shijieh/imageJ-annotate). By leveraging the broad user base and ease of installation of the software, CellScore enables researchers to obtain reliable cell counts from standard microscopy images with minimal specialized equipment. Although the workflow has only been tested with three microalgae species of similar morphologies, the pipeline balances automation with user control and includes practical troubleshooting strategies to facilitate adoption by new users. Thus, CellScore has the potential to be used broadly across other species, however, broader validation across diverse morphologies is required. Overall, CellScore provides a cost-effective, user-friendly, and reproducible solution for routine microalgal cell quantification in both laboratory and applied settings.

**Supplementary Information:**

The online version contains supplementary material available at https://doi.org/10.1007/s44307-026-00131-w.

## Introduction

**Fig. 1:**
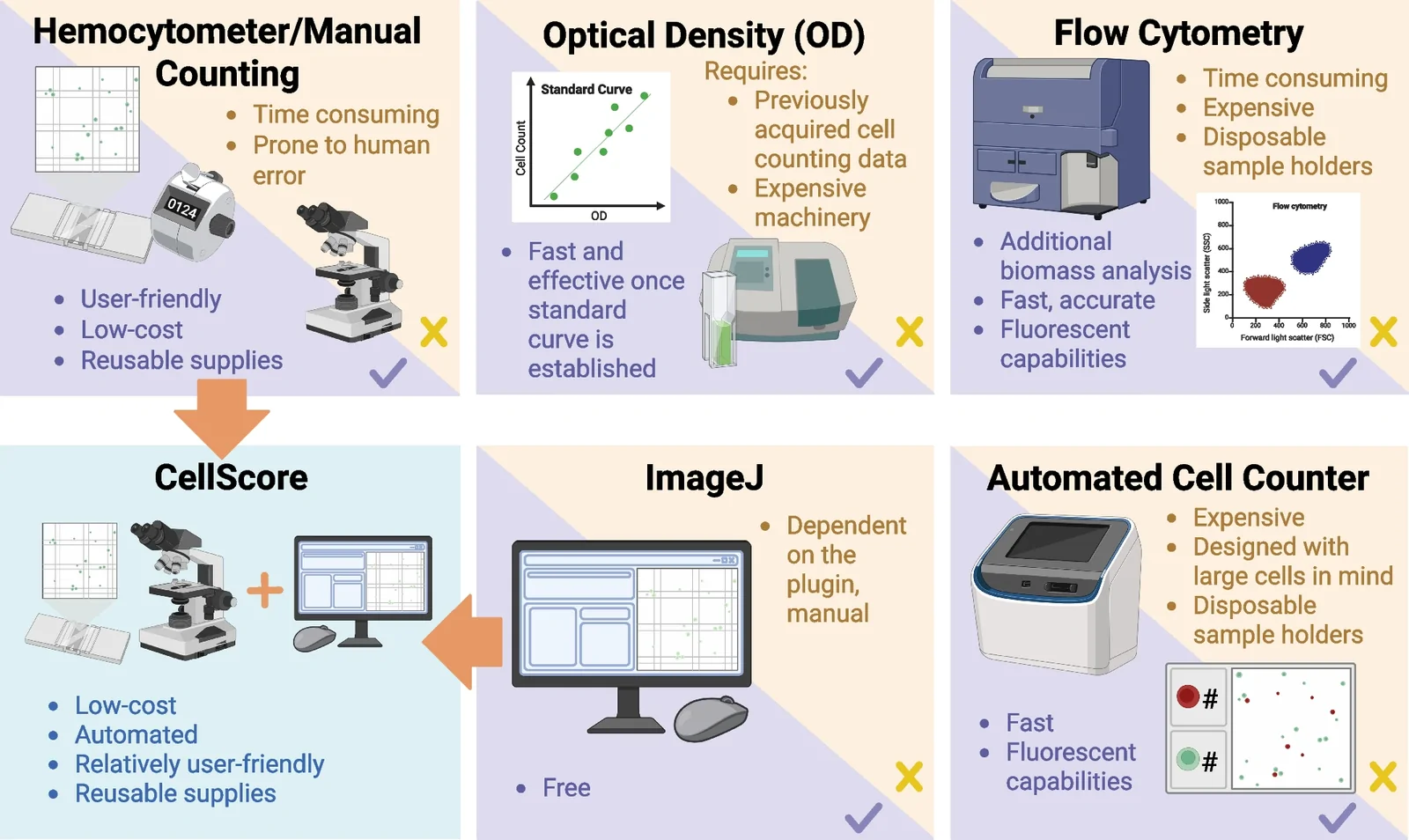
To obtain cell counts, multiple methods can be utilized. However, each has its own advantages and drawbacks. CellScore utilizes two of these methods in tandem to deliver a quick, accurate cell counting technique

Microalgae are photosynthetic microbes lauded for their productivity. These productivity rates have been utilized to design frameworks for products such as nutraceuticals, pharmaceuticals, cosmetics, and biodiesel (Sun et al. 2003; Coelho et al. 2021; Grahl et al. 2020; Xia et al. 2022; Garcia et al. 2024). In turn, various microalgae-based companies and research projects rely upon microbial capabilities. During microalgal cultivation, the density of liquid cultures is key in determining the organisms’ growth stage and its resulting biomass productivity. Therefore, high densities equate to high cell count. Currently, various methods exist to obtain cell numbers (Fig. 1, Table 1).

**Table 1:** A summarization of cell counting methods

Cell Counting Method	Device	Advantages	Disadvantages	Approximate Cost	References
Hemocytometer		- User-friendly - Cost - Reusable	- Potential of human error - Time consuming	$	-
Optical Density (OD)	Biochrom Libra S6	- Fast, accurate	- Cost - Requires linear regression models	$$	(Pahija and Hui 2019)
	UV–Vis Spectrophotometer Kasuaki IL-593-S	- Fast, accurate	- Requires linear regression models	$$	(Pessoa et al. 2025)
	Genesys 6 Spectrophotometer	- Fast, accurate - Good method for biomass estimation	- Cost - Requires linear regression models - Results can be dependent on environmental conditions	$$	(Nielsen and Hansen 2019)
	Shimadzu UV-1601 Double Beam	- Fast, accurate	- Cost	$$	(Okomoda et al. 2021)
Flow Cytometer	guava® Flow Cytometry easyCyte™ 8HT	- Fast - Multiple analysis modes	- Cost - Requires disposable, non-specialized microplates/sample tubes	$$-$$$	(Malych et al. 2022)
	BD FACS Canto II Flow Cytometer	- Fast, accurate results - Multiple analysis modes - Ideal for monitoring culture growth	- Cost - Requires specific disposable sample tubes	$$$	(Chioccioli et al. 2014)
	BD FACSAria Flow Cytometer			$$$	
	Bio-Rad BRYTE HS Flow Cytometer	- Fast - Can detect chlorophyll *a* fluorescence - No bead calibration required	- Cost - Older model	$$$	(Franklin et al. 2001)
	High-throughput, portable imaging flow cytometer	- Low cost - Unharmful to analyzed cells - No treatment steps, does not create pollutants	- No fluorescent capabilities - Images all entities in a sample	$$	(Göröcs et al. 2020; Işil et al. 2021)
Automated Cell Counter	Invitrogen™ Countess™ Automated Cell Counter	- Fast, accurate results - Can detect fluorescence	- Cost - Requires disposable, specialized chamber slides	$$$	(Takahashi 2018)
	Denovix CellDrop BFx	- No requirement for specialized, disposable supplies - Fast - Small sample requirement (≥ 2.5 uL)	- Cost	$$$	(DeNovix, https://www.denovix.com/products/celldrop/, accessed on 2026 Jan 22)
	Denovix CellDrop FLxi	- No requirement for specialized, disposable supplies - Fast - Small sample requirement (≥ 2.5 uL) - Fluorescent capabilities	- Cost	$$$	

$ (≤ $300) $$ ($300-$4,000) $$$ (≥ $4000)

Firstly, there are hemocytometers. As a counting method, it utilizes instruments many laboratories may have access to, such as a microscope, and low-cost materials like a hemocytometer and the accompanying accessories to load and examine it (Camacho-Fernández et al. 2018). Since users are required to manually count each cell, time requirements are high (Cadena-Herrera et al. 2015). Additionally, there is the risk of introducing human error, dependent on factors including the expertise level of the individual counting (Cadena-Herrera et al. 2015; Borisova et al. 2025). Despite these disadvantages, Overall, the hemocytometer can provide the highest accuracy compared to other automated methods like flow cytometry (Camacho-Fernández et al., 2018). To make use of its accuracy, hemocytometry can be accompanied with other methods to address its downsides.

The cell counts obtained with a hemocytometer are used in tandem with the optical density (OD) method. Cell count can be utilized to create a standard curve with absorbance readings, and thus the correlation between OD measurements and cell count can be used to calculate an approximate cell total. The OD method requires a spectrophotometer and its accompanying supplies, and hemocytometry supplies, therefore bringing additional costs. Furthermore, the absorbance measurements taken at different wavelengths will not record the same readings (Nielsen and Hansen 2019; Pessoa et al. 2025). Especially when comparing the two common wavelengths for microalgae cell count measurements of 680 nm and 750 nm (Sarrafzadeh et al. 2015; Nielsen and Hansen 2019; Pessoa et al. 2025). Both wavelengths are popularly utilized; however, at a wavelength of 750 nm, absorbance results are not influenced by the presence of chlorophyll *a,* but may be more indiscriminate in their measurements to include non-microalgae cells or particles within the sample (Nielsen and Hansen 2019). OD measurements can also vary for cells experiencing environmental stress, as it impacts the organisms' absorbance signal (Nielsen and Hansen 2019). To utilize OD measurements as a cell counting method requires careful considerations, including accuracy and precision confirmations before proceeding (Sarrafzadeh et al. 2015).

Another alternative measurement technique, a technique comparable to manual counting, is flow cytometry (Günerken et al. 2017). In addition to cell count data, flow cytometry can be utilized to simultaneously analyze microalgae biomass, membrane potential, and viability (Franklin et al. 2001; Chioccioli et al. 2014; Galotti et al. 2020; Park et al. 2023; Wang et al. 2023). However, if only cell count data is required, the technique is expensive, time consuming, and requires a level of expertise (Sarrafzadeh et al. 2015). Furthermore, users need to determine their own best practice for their flow cytometry protocol, as an ideal method can differ between species (Čertnerová and Galbraith 2021). Instead, users can opt for dedicated counting devices.

There are several automated cell counters available on the market. Some models have additional functions, such as fluorescence detection abilities for chlorophyll analysis (DeNovix, https://www.denovix.com/products/celldrop/, accessed on 2026 Jan 22). However, users are required to have access to or purchase the instrument, leading to extra considerations or expenses. Furthermore, the efficacy of these devices has been tested with larger cells from animal and human subjects (Takahashi 2018). For smaller microalgae cells, accuracy inquiries must be pursued to determine a device’s efficiency (Takahashi 2018). Additionally, these systems can rely on expensive, specialized supplies like counting chamber slides. As with other cell counting methods, automated cell counting devices are accompanied with their own disadvantages.

Cell counting programs for small-celled organisms can potentially address the downsides of common techniques. For instance, mouse microglia cells, around 12 µm in size, can be accurately counted with a training-based software (Kataras et al. 2023). For microalgae, training as an additional step may pose issues as species can range in morphology, and may require a more intensive training phase before use. Furthermore, overtraining is possible (Kataras et al. 2023). For users working with multiple microalgae species, programs requiring training steps may not be ideal.

More popularly, ImageJ, a free-to-use program, is a staple for cell counting purposes. ImageJ’s Particle Analysis plugin has successfully utilized Raspberry Pi in a closed system to count the filaments of *Spirulina platensis* with no significant differences to results obtained from manual cell counting and at faster rates (Tolentino et al. 2021). ImageJ was also utilized to obtain cell counting data for *Chlorella vulgaris* (Fekete et al. 2024). Although the method was reported to be both efficient and accurate, no comparisons were discussed between ImageJ and manual counting, as the research focus encompassed another alternative method for algae density estimation (Fekete et al. 2024). Furthermore, ImageJ plugins have been developed for the purpose of automating hemocytometer counting. The Cell Concentration Calculator (CCC) functions as one such method (O’Brien et al. 2016). A comparison between CCC and the manual Cell Counter function on ImageJ reveals that CCC provides comparable accuracy (O’Brien et al. 2016). Since the accuracy comparisons were done using images of human cells, further tests are required for the plugin’s functionality involving microalgae species (O’Brien et al. 2016). Beyond ImageJ, separate programs have been developed to collect cell count data.

Specialized software programs have potential in or were tested for microalgae cell counting. Deep learning algorithms such as YOLOv5 have also been observed to effectively count a species of microalgae, *Protoceratium reticulatum*, with an approximate 97% precision and about a 98% recall (Proença et al. 2022). Furthermore, the newest model, YOLOv8, was reported to provide accurate counting data (Thiviyanathan et al. 2025). However, the same version had slight fluctuations in precision and accuracy depending on the number of cells within the image (Thiviyanathan et al. 2025). Additionally, limitations of the YOLO algorithm include diversifying the image decks utilized for training (Thiviyanathan et al. 2025). *Tetraselmis gracilis* samples could also be reliably measured utilizing the Laboratory of Plankton Systems (LAPS) Frame Rhythm (FR) method (Gomes et al. 2022). A technique comparable to counting chambers, with a limit of agreement of 95%, with a 14-fold decrease in sample processing time, requiring ten minutes to count 24,000 cells (Gomes et al. 2022). FR is a technique with the capability to sort cells based on morphological characteristics (Gomes et al. 2022). To use this method with smaller microalgae cells, further studies are required (Gomes et al. 2022). A MATLAB-based automated cell counter utilized image-processing to accurately and precisely detect viable and non-viable human cervical carcinoma (HeLa) cells (Chen and Chiang 2024). However, the maximum tally was around 250 cells (Chen and Chiang 2024). For microalgae and high-density cell cultures, the efficacy of the workflow has not been elucidated (Chen & Chiang, 2024). Presently, most existing software programs have not been explored for counting small microalgae cells at higher densities.

With the current technologies available, there remain several limitations in obtaining automated microalgae cell count data. These limitations include non-microalgae organisms as the focus of the counting method. Additionally, although there are programs available to quickly record cell count, they have not yet been officially tested with smaller microalgae cells at high densities. Furthermore, some of these workflows require additional supplies, such as expensive machinery or disposable materials. Lastly, for traditional cell counting methods, there are clear disadvantages such as time requirements, cost, and user-friendly capabilities. As such, we aim to create a cell counting program to address these issues. A user-friendly program with the ability to quickly and accurately detect microalgae, utilizes supplies a lab may already have (computer, microscope, hemocytometer, and camera), does not require a lengthy user training period, and has been tested with high-density cultures (> 2,500 cells).

## Materials and methods

### Microalgae cultivation

Six strains of *Chlamydomonas reinhardtii* were obtained from the Chlamydomonas Resource Center (Minnesota, USA): Sager’s basic wild-type strain 21 gr (Sager 1955), wall-deficient mutant cw15 (Davies and Plaskitt 1971), wall-deficient starchless mutant sta6 (Li et al. 2010), wall-deficient starchless mutant sta7 (Posewitz et al. 2004), wall-deficient flagellar mutant pf18-, cell-walled flagellar mutant fla3-1 (Mueller et al. 2005); in addition, one strain of *Chlorella sorokiniana* KAS908 (Kuehnle et al. 2015) and one culture of *Micractinium reisseri* PRAS were inoculated into liquid tris–acetate phosphate (TAP) medium. The new inoculants were left on a shaker at 120 rpm with a light intensity of 80 µmol m^−2^ s^−1^ for two days and subsequently fed the same media every two days for a duration of a week.

### Image collection

Hemocytometer images were taken of each strain at no, 2×, 3×, and 4× dilution after 48 to 72 hours of growth or until the culture achieved stationary phase. The stationary growth phase was confirmed through absorbance measurements, with a VWR UV-1600PC, UV/Visible Spectrophotometer, once the absorbance readings plateaued. Each image depicted the Rs’ Science Neubauer hemocytometer with 10 µL of sample from each culture. The hemocytometer was viewed with a Nikon ECLIPSE Si trinocular microscope at 10×, and images were taken with a RisingCam Industrial Digital Camera E3IS PM 20 PM camera. The illumination and exposure of the images were not standardized; however, all images utilized a level of illumination to effectively view each cell and the accompanying hemocytometer lines. All images were saved as JPEG files. The images of *C. reinhardtii* and *C*. *sorokiniana* at 3× dilution, and images of *M*. *reisseri* at 4× dilution were utilized for counting. The users of the manual and automated counting methods were not blinded, and images were processed sequentially.

### Manual counting

The images were initially counted manually with two methods. The first method involved manual counting with a clicker. The second counting method employed the Cell Counter plugin on ImageJ/Fiji (version 1.54, Fiji; RRID:SCR_002285) (Schindelin et al. 2012). The latter method was referenced as the most accurate counting method or control variable (Schindelin et al. 2012). Error rates were calculated with the following equation, where the experimental cell count denoted the cell count obtained from either, clicker counting (manual), CellProfiler (automated), ImageJ (automated), or CellScore (automated), respectively:

1. Error rate = |(Experimental Cell Count-ImageJ (manual) Cell Count)/ImageJ (manual)|*100

### Automated cell counting

To test an established counting software with hemocytometer images, Ilastik (version 1.4.1rc2; RRID:SCR_015246), followed by CellProfiler (version 4.2.8; RRID:SCR_007358), was utilized as an automated cell counting test (Berg et al. 2019; Stirling et al. 2021). Ilastik served as a segmentation tool, removing background noise and separating aggregating cells. CellProfiler performed the counting. The users of both Ilastik and CellProfiler did not receive prior training or have familiarity with the software combination. Additionally, the native ImageJ tool, Analyze Particles, was utilized as an experimental automated cell counting method. The images were converted into the 8-bit format and ran through the same threshold values utilized for CellScore (Table 2). To set up the Analyze Particles tool, size and circularity ranges also followed the same values utilized for CellScore (Table 2).

**Table 2:** The size, circularity, and threshold values utilized for three microalgae species

Species	Strain	Size	Circularity	Threshold
*Chlamydomonas reinhardtii*	21 gr	20-Infinity	0.20–1.00	0, 140
	cw15	50-Infinity	0.30–1.00	0, 150
	sta6	50-Infinity	0.20–1.00	0, 150
	sta7	50-Infinity	0.20–1.00	0, 150
	pf18-	50-Infinity	0.20–1.00	0, 156
	fla3-1	50-Infinity	0.20–1.00	0, 150
*Chlorella sorokiniana*	KAS908	5-Infinity	0.20–1.00	0, 165
*Micractinium reisseri*	PRAS	5-Infinity	0.20–1.00	0, 165

### Software description

CellScore utilizes ImageJ’s Analyze Particles function. This function identifies objects with specific size and circularity criteria on a binary, or black and white, image. To maximize the effectiveness of the cell counting, a series of pre-processing steps for the colored hemocytometer images was conducted, including conversion to grey scale, binarization (using a manually selected threshold to remove background noise), filling holes in the cells (mostly generated by an aggressive threshold from the last step), and separating overlapping cells. All the pre-processing steps are achieved using ImageJ built-in plugins (ImageJ Docs 2022). The last step involves applying the Analyze Particles function to the pre-processed image, which will count the number of cells that satisfy the selected cell size and circularity criteria. A trial-and-error procedure may be necessary to find the appropriate cell size and circularity values.

**Fig. 2:**
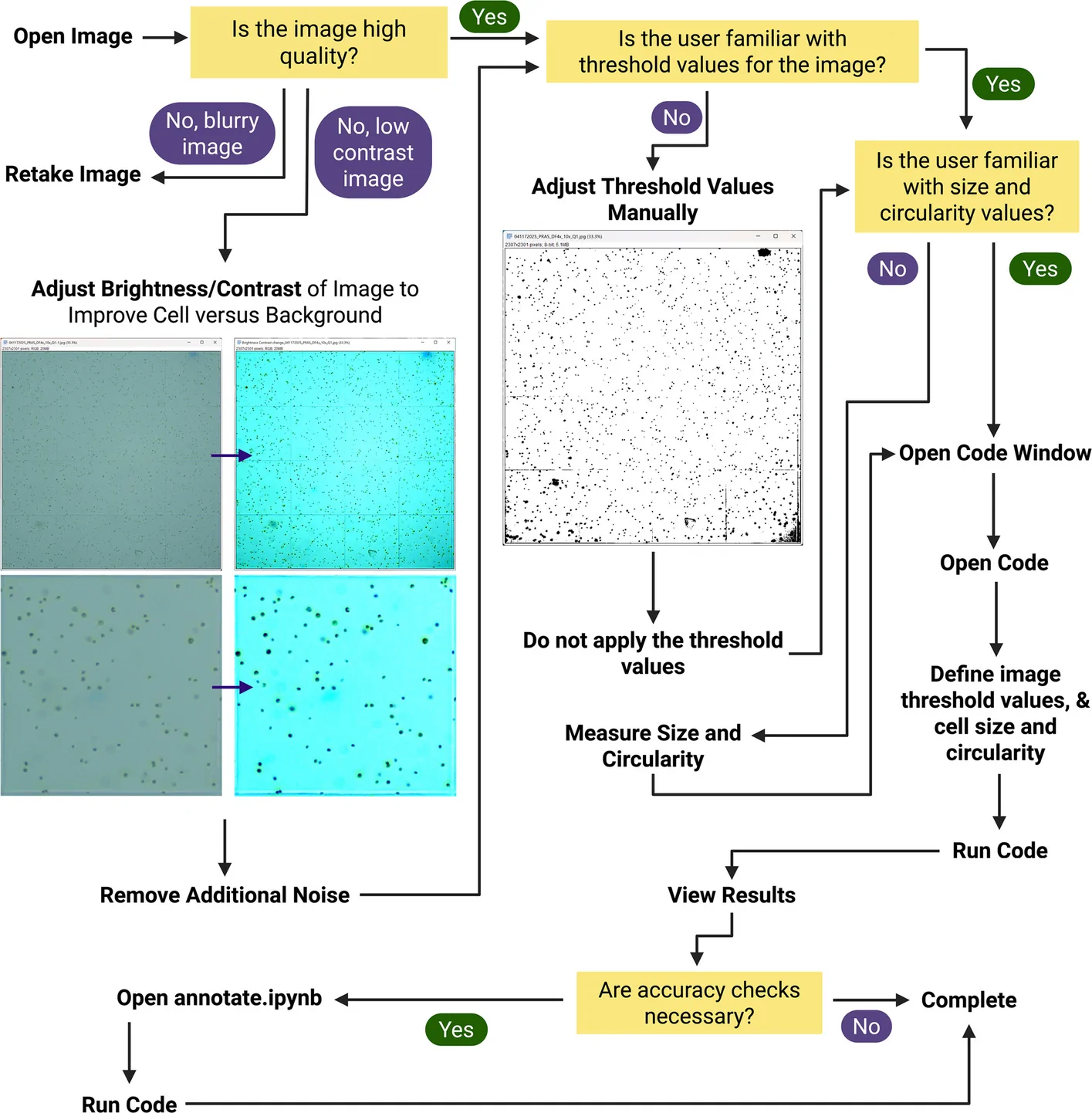
Images may require more processing time dependent on the quality of the image and the user's familiarity with the ImageJ software. Where additional steps to address these factors can be implemented. The optional, “Adjust Brightness/Contrast,” step can be completed through selecting Image > Adjust > Brightness/Contrast, where the user can determine the optimal brightness and contrast settings to increase the difference between cell and background. Following the previous step, the user is prompted to, “Remove Additional Noise,” achievable through selecting, Image > Color > Split Channels. ImageJ will be prompted to split the image into three channels. The user is tasked with selecting the window with the clearest contrast between cell and background. If the user is unfamiliar with threshold values, they are tasked to, “Adjust Threshold Values Manually,” through selecting, Image > Type > 8-Bit > Image > Adjust > Threshold. Once an ideal threshold range is achieved, users must use the same range within the ImageJ macro of CellScore, not apply the range to the image. If the user is unfamiliar with ImageJ, there are native functions to measure size and circularity. Both of which are achievable through the suggested workflow, select the Freehand tool and outline the cell > Analyze > Set Measurements > ensure both, “Area,” and “Shape Descriptors,” are selected > Analyze > Measure. The workflow prompts a window to open, containing pixel measurements of the selected object. Users are recommended to follow this protocol for both the smallest cell, and the least circular. Furthermore, if other objects in the image are present, such as cell debris, measurements should also be taken for the largest cell and most circular. Users are also asked to ensure the range for both these values are slightly adjusted to account for cells that are smaller or larger, less or more circular, or both than the cells chosen. Additionally, the user must edit the save path of the results in the macro to accurately define where the file is recorded. After the code has been run, there is an optional Python method to overlay the annotations made by CellScore over the original image to ensure cell counting values have been accurately obtained

Circularity is defined by the equation: 4π*area/perimeter^2^ (imagej.net 2014). Whereas size encompasses an object’s width and height (imagej.net 2014). To determine size and circularity values, the user is recommended to use the following steps (Fig. 2; Table 2):

1. Open the image (File > Open)
2. Select the Freehand tool
3. Identify the smallest cell
4. Outline the cell
5. Measure size and circularity (Analyze > Set Measurements > select “Area,” and “Shape Descriptors,” for size and circularity values, respectively > Analyze > Measure)
6. Repeat steps 2–5 for the least circular cell. If debris is present in the image, it is recommended to repeat the same steps for the largest and most circular cell to specify the ranges for these characteristics.

The following describes the steps to use CellScore (Fig. 2):

1. Open the image (File > Open)
2. Open code window (Plugins > Macros > Startup Macros)
3. Open code (within the Macros window, File > Open > select the “auto_run.ijm” file)
4. Define the image’s threshold range, cell size, and circularity, by inputting the desired ranges in the macro, and edit the save path of the file.
5. Run code (within the Macros window, select Run)
6. View results (the summarized results window records the cell count, whereas the results window records all the objects counted as a cell within the image with coordinates)
7. If accuracy checks are necessary, use the “annotate.ipynb” file.

### Statistical Analysis

The sample size, n, defines nine sets of four images of each hemocytometer quadrant. A total of 36 images were run through each counting method. Significance was determined by calculating the difference between the count obtained by utilizing the ImageJ manual function and the count obtained with each experimental method. The two-tailed Wilcoxon signed-rank test at a significance threshold (α) of 0.01 was utilized to determine the statistical significance of the deviation between the ImageJ manual count and experimental methods. The statistical analysis was performed on the cell counting values obtained from each of the 36 images and sample size varied from 34-36 dependent on the amount of non-zero values. The null hypothesis stated there was no significant difference between the counts obtained by the manual ImageJ method and the experimental method.

## Results

**Fig. 3:**
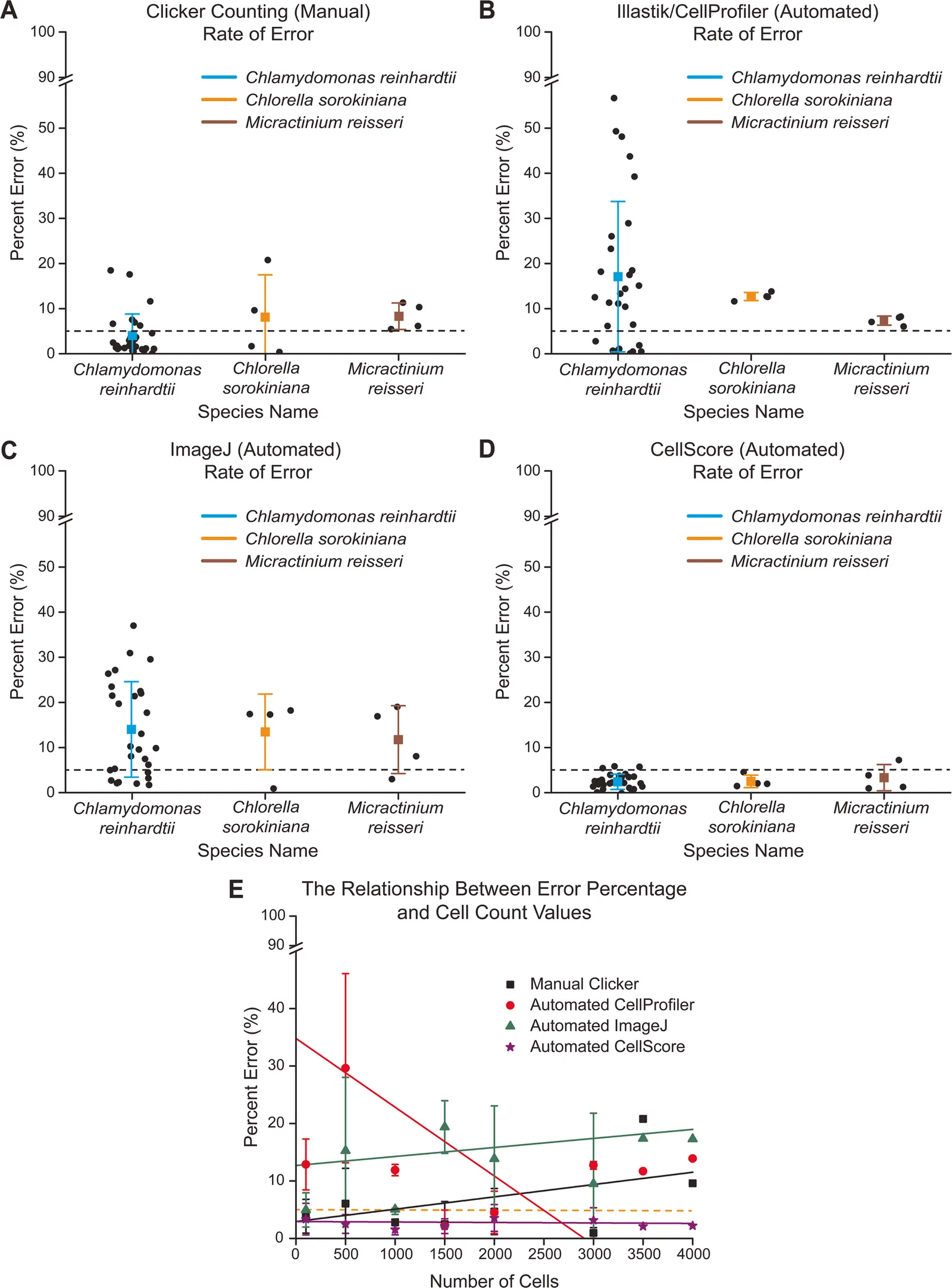
**A** The percentage of error associated with manually counting with a clicker, the black dotted line represents a 5% error threshold. Error bars represent the standard deviation (n = 9). **B** The percentage of error associated with automated counting using Ilastik and CellProfiler, the black dotted line represents a 5% error threshold. Error bars represent the standard deviation (n = 9). **C** The percentage of error associated with automated counting using the Analyze Particles function in ImageJ, the black dotted line represents a 5% error threshold. Error bars represent the standard deviation (n = 9). **D** The percentage of error associated with automated counting using CellScore, the black dotted line represents a 5% error threshold. Error bars represent the standard deviation (n = 9). **E** The graph depicts the fluctuations in error with varied cell count across the four experimental cell counting methods, the orange dotted line represents a 5% error threshold. Error bars represent the standard deviation (n = 9)

As the hemocytometer is comprised of four, four-by-four cubed areas, Fig. 3A, 3B, 3C, and 3D note the error rates associated with each four-by-four square. Generally, cell count values calculated from hemocytometers are obtained by finding the average of cells on each slide, or with four of these squares. The values noting average cell count were obtained by calculating the average of all four, four-by-four squares on the hemocytometer. Therefore, error rates are lower for the reported average values.

### Manual counting with ImageJ

The hemocytometer images were uploaded to ImageJ, where the Cell Counter plugin was utilized to individually mark each cell and concurrently tally cell count. For *C. reinhardtii* 21 gr, the manual cell count averaged to 276 cells and required 21 min to record. The mutant cw15 required nine minutes to record 120 cells on average. The images of the mutant sta6 had an average of 260 cells and required 17 min for results. Whereas *C. reinhardtii* sta7 took 94 min for an average cell count of 1608. The first culture replicate of *C. reinhardtii* pf18- had an average cell count of 1602 in the hemocytometer images and required 91 min for results. Whereas the second replicate resulted in an average cell count of 768 and required 47 min to obtain. Lastly, for *C. reinhardtii* fla3-1, 26 min were utilized for an average cell count of 425. *C*. *sorokiniana* KAS908 required 186 min for an average count of 3258 cells. The culture of *M. reisseri* PRAS, required 80 min to count an average of 1934 cells.

To obtain these average values, each quarter of the hemocytometer was utilized to calculate cell quantity.

### Manual counting with clicker

A sample of *C. reinhardtii* 21 gr had an observed average count of 295 cells, with an error of 7.0% when compared to manual counting data obtained from ImageJ’s cell counter plugin. The average count was obtained in nine minutes. A sample of *C. reinhardtii* cw15 had an average cell count of 116 with an error of 3.1%. The cell count was recorded in four minutes. For a sample of *C. reinhardtii* sta6, the average cell count, and accompanying error percentage were 232 and 10.8%, respectively. These values were obtained in eight minutes. As for *C. reinhardtii* sta7, it took 52 min to record an average cell count of 1600 cells, with an error rate of 0.5%. *C. reinhardtii* pf18- was also counted; the process utilized 53 min, with an average cell count of 1612 and 0.6% accompanying percent error. Replicate images of the strain were also obtained at the same dilution, where an observed average of 761 cells was accounted for with a 0.9% error rate. The result required 29 min to record. Lastly, *C. reinhardtii* fla3-1 took 17 min to observe an average cell count of 423 and an error rate of 0.5%. The culture of *C. sorokiniana* KAS908 had an average cell count of 2964, an error percentage of 9.0%, and required 105 min to obtain. Whereas *M. reisseri* PRAS utilized an hour of counting time to record an average cell count of 1777 cells, with an error of approximately 8.1%.

The individual error rates of each image were also aggregated. These error values consistently rose above the acceptable 5% (Benetz et al. 2006; Hammes and Egli, 2010; Huang et al. 2018; Mizuno et al. 2024) (Fig. 3A). Furthermore, the accuracy of manual counting with a clicker would decrease as the cell density increased (Fig. 3E).

### Automated counting with Ilastik and CellProfiler

**Fig. 4:**
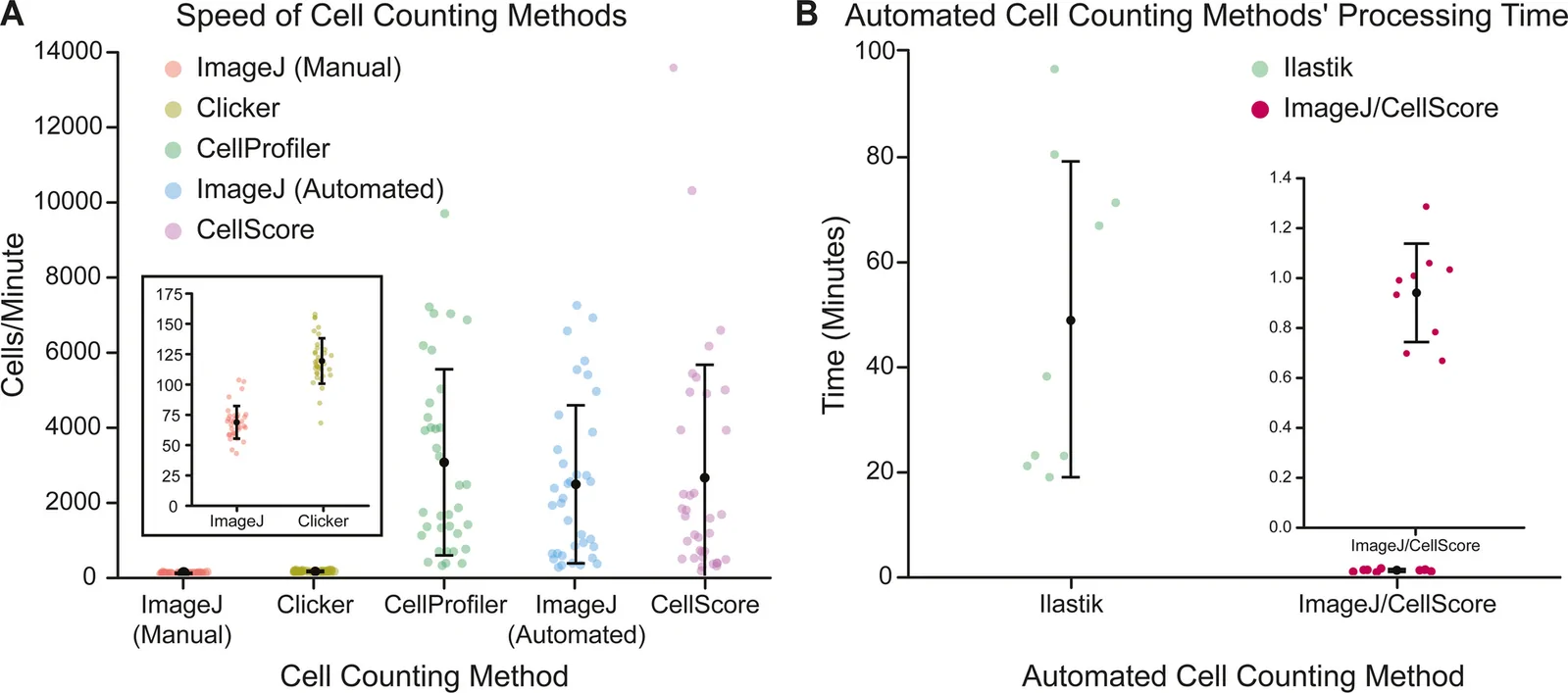
**A** The comparison of four cell counting methods revealed varied speeds overall. With ImageJ and Clicker counting, both two manual methods, their counting speed is significantly slower than the three automated methods, CellProfiler, ImageJ, and CellScore. Error bars represent the standard deviation (n = 9). **B** CellProfiler and ImageJ-based cell counting methods both require preprocessing steps to segment and measure the image, respectively. These steps are timed separately from the run time required to count each image. Error bars represent the standard deviation (n = 9)

For *C. reinhardtii* 21 gr the image processing step using Ilastik, took 95.85 min, and running the resulting image through CellProfiler required 1.3 min. This process recorded an average cell count of 411 with a percent error of 49%. The cell wall-less mutant, cw15, utilized 22.68 min to undergo Ilastik segmentation. An average cell count of 102 was recorded, requiring 1.3 min to obtain, with an error of 14%. *C. reinhardtii* sta6 employed 79.67 min for processing and measured an average of around 226 cells with an accuracy of 13%, in 1.4 min. Whereas the strain sta7, utilized 70.55 min for Ilastik segmentation and 1.7 min through CellProfiler, to record a cell count of 1597 cells with an error rate of 0.7%. The first replicate of *C. reinhardtii* pf18- images required 22.58 min to process with Ilastik. CellProfiler required 1.5 min to record an average count of 1601 with a 0.1% error rate. The second set of *C. reinhardtii* pf18- images required 20.68 min to process and 1.2 min to record an average cell count of 629 and 18% error. The fla3-1 strain utilized 66.19 min for segmentation and resulted in an average count of 512 cells with an error rate of 20% after 1.4 min. *C. sorokiniana* KAS908 required 18.55 min for the image processing step. The cell counts for all hemocytometer quadrants were measured in 1.5 min, resulting in an average of 2841 cells and a 13% error. The culture of *M. reisseri* PRAS utilized 37.65 min to process on Ilastik and 1.4 min in CellProfiler to obtain an average cell count of 2076 across the hemocytometer (Fig. 4B). The cell count data resulted in an error of about 7.4%.

The cell counts obtained from the 36 images collected tended to exceed the accepted 5% error (Benetz et al. 2006; Hammes and Egli, 2010; Huang et al. 2018; Mizuno et al. 2024) (Fig. 3B). The resulting high error and long duration of image processing may be attributed to low user familiarity with both Ilastik and CellProfiler. Whereas the accuracy of the workflow exhibits a direct relationship with cell count (Fig. 3E).

### Automated counting with ImageJ

The strain, *C*. *reinhardtii* 21 gr, ran with the Analyze Particles function took a duration of 1.79 min to record an average count of 194 cells, with an error of 29.7%. Prior to running the tool, one minute was taken to measure size, circularity, and threshold values. Whereas the mutant strain, cw15, utilized 1.28 min to measure the culture’s features, and 1.62 min to obtain an average count of 115 cells with a 3.8% error rate. A culture of the starchless variant, sta6, resulted in an average count of 227 cells, utilizing 56 s for the measurement steps, and 1.39 min to run ImageJ, followed by an error percentage of 12.6%. The culture of sta7 had taken 46 s to record measurements and 1.37 min to observe an average count of 1279 cells with an accompanying 20.5% error. The first replicate of pf18- took 1.42 min to quantify an average of 1299 cells with 18.9% error. The second replicate of pf18- took 1.43 min to tally an average of 738 cells, and an error of 3.9%. For the strain, about 40 s were utilized to record size, circularity, and threshold. Lastly, fla3-1 utilized 1.05 min for measurement and 1.51 min to record an average of 409 cells with an error of 3.8%. For the culture of KAS908 the average count was 2818 cells, employing 1.71 min, with a 13.5% error. Whereas the measurement step used approximately 42 s. PRAS, a strain of *M*. *reisseri*, required 2.07 min for an average count of 2152 cells with a 11.3% error. The previous measurement step took 1.02 min to record the cells’ attributes within the image (Fig. 4B).

The error percentages of cell counts collected with the Analyze Particles function often exceed 5% (Benetz et al. 2006; Hammes and Egli, 2010; Huang et al. 2018; Mizuno et al. 2024) (Fig. 3C). The high errors were attributed to the workflow simply utilizing one ImageJ tool without any further processing of the image such as Watershed or Fill Holes. The absent processing steps prevented the ImageJ tool from accurately identifying objects as cells. Furthermore, the accuracy of the workflow is heavily correlated with image quality, rather than cell count (Fig. 3E).

### Automated counting with CellScore

The same size and circularity measurements employed to run ImageJ’s Analyze Particles function were utilized for CellScore (Fig. 4B). Therefore, the same preprocessing time remains applicable for the automated ImageJ method and CellScore. For *C*. *reinhardtii* 21 gr, the cell counter utilized approximately 2.2 min and obtained an average cell count of 280, with an 1.3% error. The same species' mutant variant, cw15, required 1.7 min for an average count of 123 cells on the hemocytometer, thus resulting in an error of 2.9%. Images depicting *C*. *reinhardtii* sta6 counted with CellScore had 256 cells on average, resulting in an 1.5% error, utilizing 3.7 min. Whereas sta7 utilized 1.7 min to obtain a cell count of 1647 with a 2.4% error rate. The first set of pf18- took approximately 1.0 min to gather an average cell count of 1639, with a 2.3% error. Whereas the second replicate of the same strain required around 2.4 min for an average cell count of 785, accompanied by an error rate of 2.3%. The last mutant of *C*. *reinhardtii*, fla3-1, needed 1.6 min to obtain an average cell count of 428 cells with an 0.6% error. For *C*. *sorokiniana* KAS908, an average count of 3194 cells was recorded in 1.6 min with an error percentage of 2.0%. *M. reisseri* PRAS utilized a total of approximately 5 min for an average cell count of 1965 with a 1.6% error.

With CellScore, the error percentages of each individual image would hover around or remain lower than 5% (Benetz et al. 2006; Hammes and Egli, 2010; Huang et al. 2018; Mizuno et al. 2024) (Fig. 3D). Out of the three experimental counting methods analyzed, CellScore provides relatively consistent accuracy across several cell quantities (Fig. 3E).

### Wilcoxon Signed-Rank Test

The Wilcoxon signed-rank test revealed with α = 0.01, the manual clicker, automated Ilastik/CellProfiler, and automated CellScore method had a greater test statistic (W) value. After omission of the zero values, each method had n = 35, 36, and 34, respectively. Therefore, the values failed to reject the null hypothesis, and there was no statistically significant difference detected. The sole experimental measurement method with a significant difference was automated ImageJ, with n = 36. The W value was smaller than the critical value, leading to rejection of the null hypothesis.

## Discussion

In comparison to other techniques, CellScore has drastically improved upon the counting speed of existing manual counting techniques and performs similarly to existing automated methods (Fig. 4A). To confidently compare the accuracy of CellScore with existing cell counting programs like CellProfiler, further elucidation is required, as a more experienced user base may be able to obtain results with lower error rates than those depicted in Fig. 3B. Although, statistical analysis has revealed the counting values obtained by CellProfiler are not significantly different than the values obtained through manual ImageJ counting. In terms of accuracy, all methods, barring the ImageJ automated technique, were not significantly different than the control manual ImageJ counting method. Through analysis of both speed and accuracy, CellScore is a great alternative to the existing cell counting workflows.

**Fig. 5:**
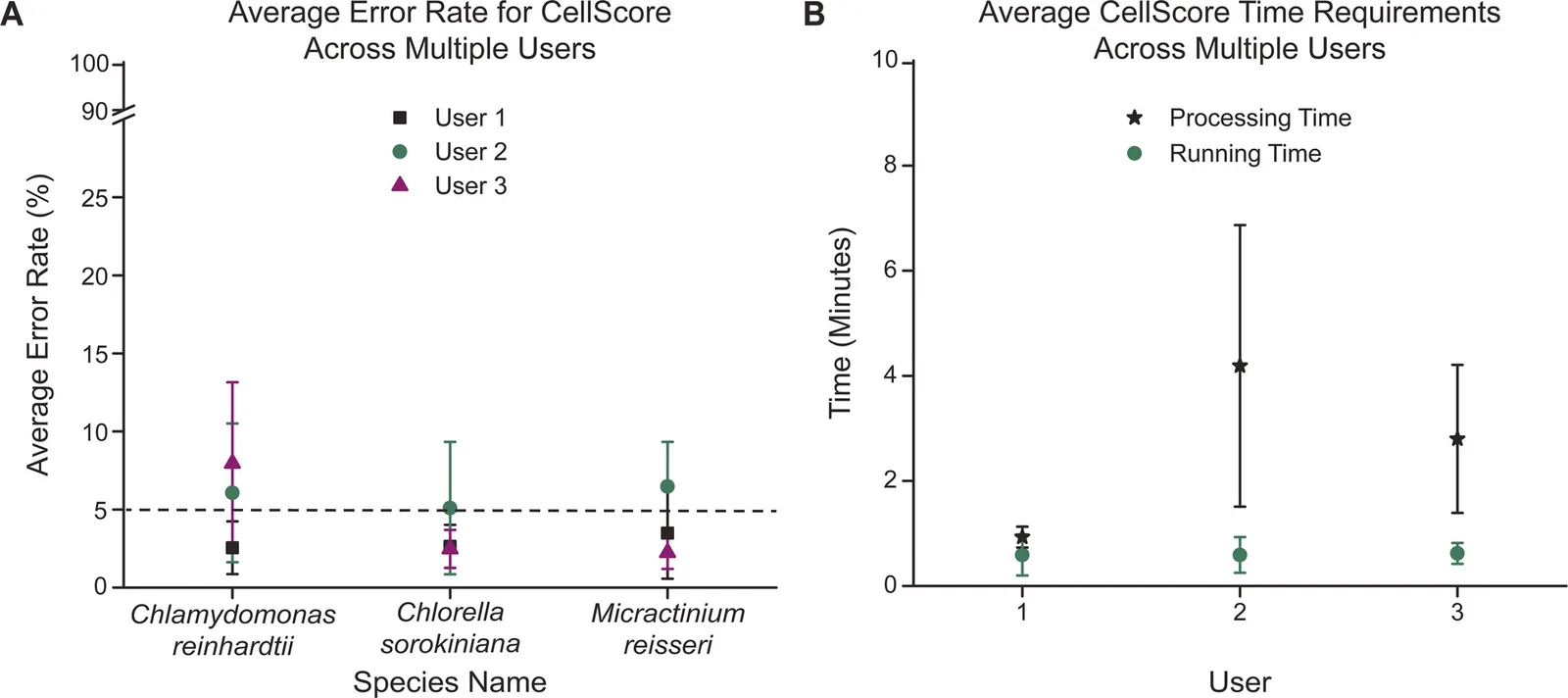
The rate of error and time requirements of CellScore across multiple users. Users 2 and 3 had no previous experience in running CellScore prior to this experiment. **A** The error rates of counting three microalgae species with CellScore obtained from three different users. The black dotted line represents the acceptable 5% error threshold. Error bars represent the standard deviation (n = 9). **B** The average one-time processing cost of CellScore required for each user and the required running time of the code. Error bars represent the standard deviation (n = 9)

The length of the processing period utilized to find the ideal settings for each image set can vary depending on image quality and prior user experience with the code (Figs. 4B and 5B). For those new to the ImageJ software, there are also several steps to assist with building familiarity (Fig. 2). Since the provided code requires the definition of the threshold range, the user can first open their image on ImageJ and manually adjust thresholding. The same threshold range used to successfully edit the image to highlight the cell, as white, and define the background, as black, can be input into the macro. From there, images with similar values can utilize the same thresholding values for cell counting purposes. If the desired image struggles with thresholding, enhancing the contrast and brightness of the image may assist with determining the range. Wherein ImageJ, the native function Brightness/Contrast, can be used to define a new range for brightness, contrast, or both. Furthermore, size and circularity definitions can also be obtained through manual measurement methods for new strains, as these characteristics can vary.

It is highly recommended for the user to familiarize themselves with their species’ size and circularity characteristics. Size is comparably simpler to determine ImageJ houses native functions to determine a cell’s area. In images free of debris, only the smallest cell’s measurement is required. Circularity can take longer to define. Since circularity is defined formulaically, the user must identify cells on each end of the spectrum: a cell that appears nearly perfectly circular and one that does not. If the image is free of debris, only the latter measurement should be obtained. Generally, gaining familiarity with CellScore, depending on the quality of the image, can take upwards of ten minutes or more (Fig. 5B). However, the timing can vary depending on the user (Fig. 5B). Some automated methods first require training the software; thus, these programs require additional time to initially run, even with an experienced user. Training also molds the respective software to specific morphologies, and will not apply broadly to many microalgae species. Whereas CellScore does not require training, which improves its user-friendliness and increases its potential applications. 

To further determine ease of use, two users with no prior experience were tasked with using CellScore to count the same set of images. They were able to obtain relatively accurate results, below a 15% error rate, utilizing less than an average of eight minutes dedicated for processing each image and less than a minute to run those images (Fig. 5A, B). The CellScore workflow only allows the customization of three categories, meaning the proficiency requirements of the user are low.

**Fig. 6:**
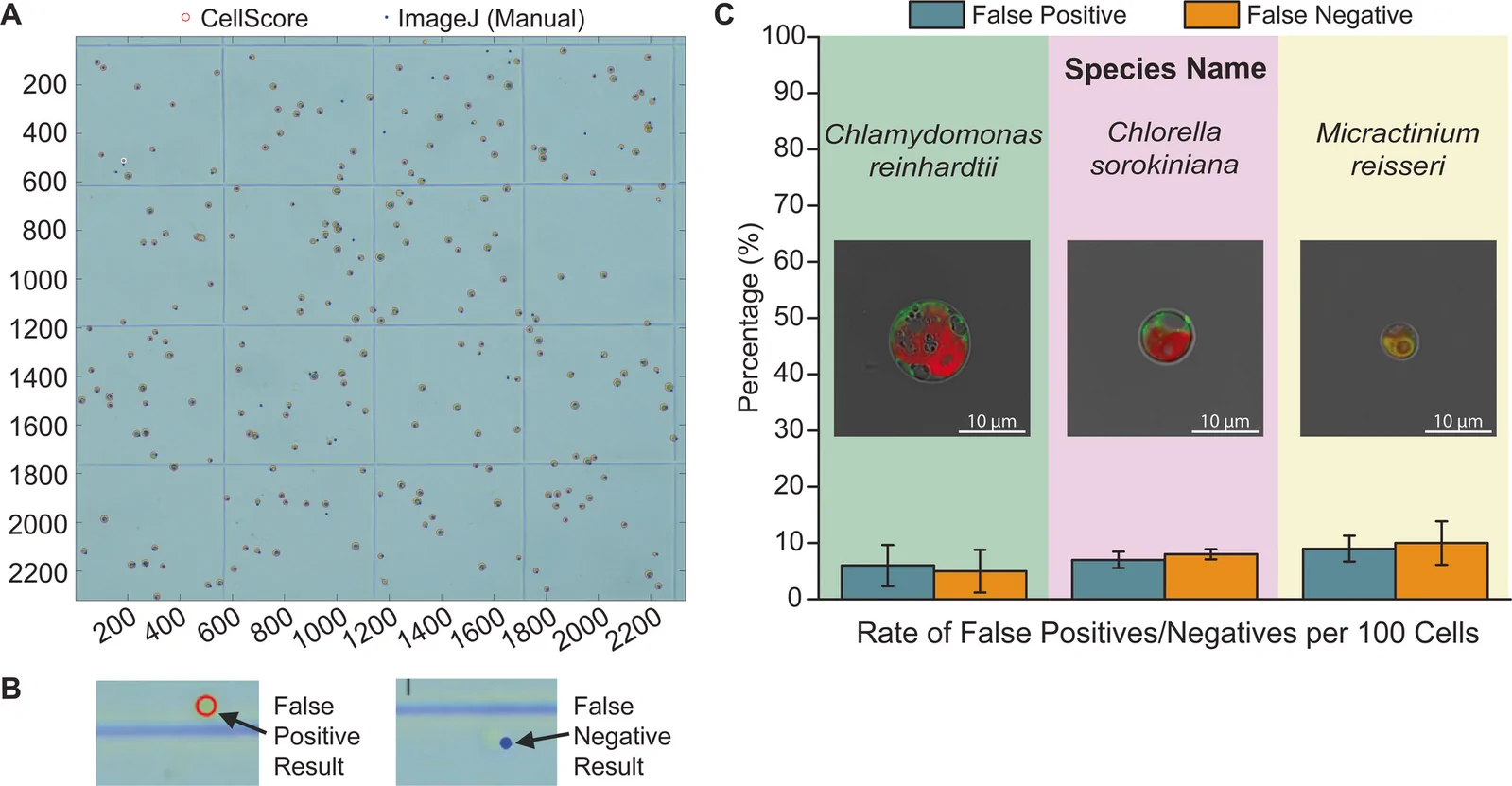
False positive and negative results from CellScore. **A** A depiction of the accuracy check code run on MATLAB, overlaying data points from the manual ImageJ and CellScore methods. **B** False positive results stem from CellScore identifying objects unmarked by the manual ImageJ software. False negatives result from the automated counter failing to identify cells. **C** A graphical representation of the percentage of false positives and negatives for each species cultivated. Error bars represent the standard deviation (n = 9). Each species is matched with a cell depicted using confocal microscopy. In each image, the cell is stained with BODIPY to roughly highlight lipids within the organism in green and the chlorophyll autofluorescence is highlighted with red

Furthermore, due to the nature of ImageJ, as a free, open-source software, CellScore is accessible to most users. However, accuracy checks were initially run on MATLAB (Version 25.2 (R2025b); RRID:SCR_001622) (The MathWorks 2026) (Fig. 6A). A code formatted with Python has been provided with the CellScore workflow on a GitHub repository to analyze the macro’s accuracy. The Python code overlays manual ImageJ annotations with the automated annotations. Without it, accuracy can still be roughly analyzed through the annotated image provided by the ImageJ software after counting has concluded.

The results from CellScore are extremely promising, as accuracy checks for false positive and negative annotations revealed an average of six cells per 100 cells are counted falsely positive, and five cells per 100 cells are recorded as a false negative (Fig. 6B, C). However, most of the false positive annotations were due to errors in identifying hemocytometer lines as cells due to the threshold range. For improved results, more dedication towards enhancing the threshold values was required. However, issues with misidentifying hemocytometer lines as cells can be mitigated by forgoing the use of a hemocytometer, as usage of the slide is optional. The hemocytometer simply allows more accurate calculations of cell concentration within the liquid culture sample. Generally, users should prioritize finding an ideal threshold range for their desired image and microalgae strain. Size and circularity options are secondary if the image has been properly processed to only highlight cells over the background. As thresholding can get rid of extra cell debris or other unwanted objects. Therefore, depending on the data to be collected, users should also customize the workflow to fit their requirements.

## Limitations & future perspectives

There are additional utilities currently unexplored for CellScore. A desirable function for the software is counting and differentiating stained from non-stained cells. Currently, it is possible to use machine learning techniques for the purpose of identifying dead microalgae cells (Reimann et al. 2020). However, for cells dyed with trypan blue, used to tally viable and non-viable cells, native ImageJ functions can be used to highlight stained and non-stained cells to quickly and accurately count cells of each group. Thus, one additional application of CellScore may lie in automating the differentiation and counting of stained and non-stained cells. There is also interest in the automated quantification of fluorescently labeled images (Lojk et al. 2015). With the current functionality of CellScore, it may be possible to diversify its usages to encompass fluorescence detection (Lojk et al. 2015). If CellScore were able to process fluorescent images, it could assist with analyzing confocal imaging results to quantify cells with specific fluorescent signals of varied brightness. Therefore, the potential applications of CellScore remain broad for microalgae.

The focus remained on microalgae cell counting; however, it may be possible to diversify CellScore's current species pool to include a variety of microbes. Future studies can explore the workflow’s efficacy with diverse microbes, like bacteria and yeast, through the pursuit of extra accuracy checks. Additionally, due to the cell counter's ability to define circularity of the pixels, it may also be possible to utilize the software for aggregate cultures containing organisms with varied morphologies. With further elucidation, CellScore may be applied to other industrial or research applications involving microbes in the future.

Although three microalgae species are highlighted, their morphology remains similar. Thus, additional tests need to be conducted for microalgae species varying in shapes and sizes. Previous endeavors to create an effective counting method have been successful. However, regardless of the counting method, those currently available do not address all existing limitations yet. Current cell counting methods require improvement in areas including cost, time requirements, accuracy for smaller cells, and user-friendliness. For the latter, the characteristic of software user-friendliness is subjective. However, with the resources available, CellScore has an approachable learning curve. Regarding the former characteristics, the ImageJ-based method makes use of an open-source, free software. Lastly, with reasonable computing capacity available to the user, counting data from microalgae cultures can be gathered quickly.

## Supplementary Information

Supplementary Material 1. A visual aid to run CellScore.

Supplementary Material 2. The Python code used after running CellScore to ensure the annotations follow annotations made by the manual Cell Counter plugin in ImageJ.

Supplementary Material 3. The CellScore macro to input into ImageJ/Fiji.

## Data Availability

All data generated or analyzed during this study are included in this published article.
