# Supplementary material for: CellScore: an open-source image-based pipeline for automated cell quantification in microalgae
Source: Adv Biotechnol (Singap). 2026 Nov 9;4(4):46. doi: 10.1007/s44307-026-00131-w (PMC13748195; doi:10.1007/s44307-026-00131-w)
Supplement: Supplementary file 1 [file 44307_2026_131_MOESM1_ESM.pdf]

Cell Counter Workflow

First, download ImageJ.

Protocol:
1. Open Image, File → Open → select your image

If applicable, preprocessing step:
1. Image → Adjust → Brightness/Contrast
   a. Adjust brightness and contrast until cells are defined
2. Image → Color → Split Channels
   a. Select image where cells are clearly defined

Otherwise, skip to step below:
1. **Run ‘auto_run.ijm’**

To check accuracy:
1. Run ‘annotate.ipynb’

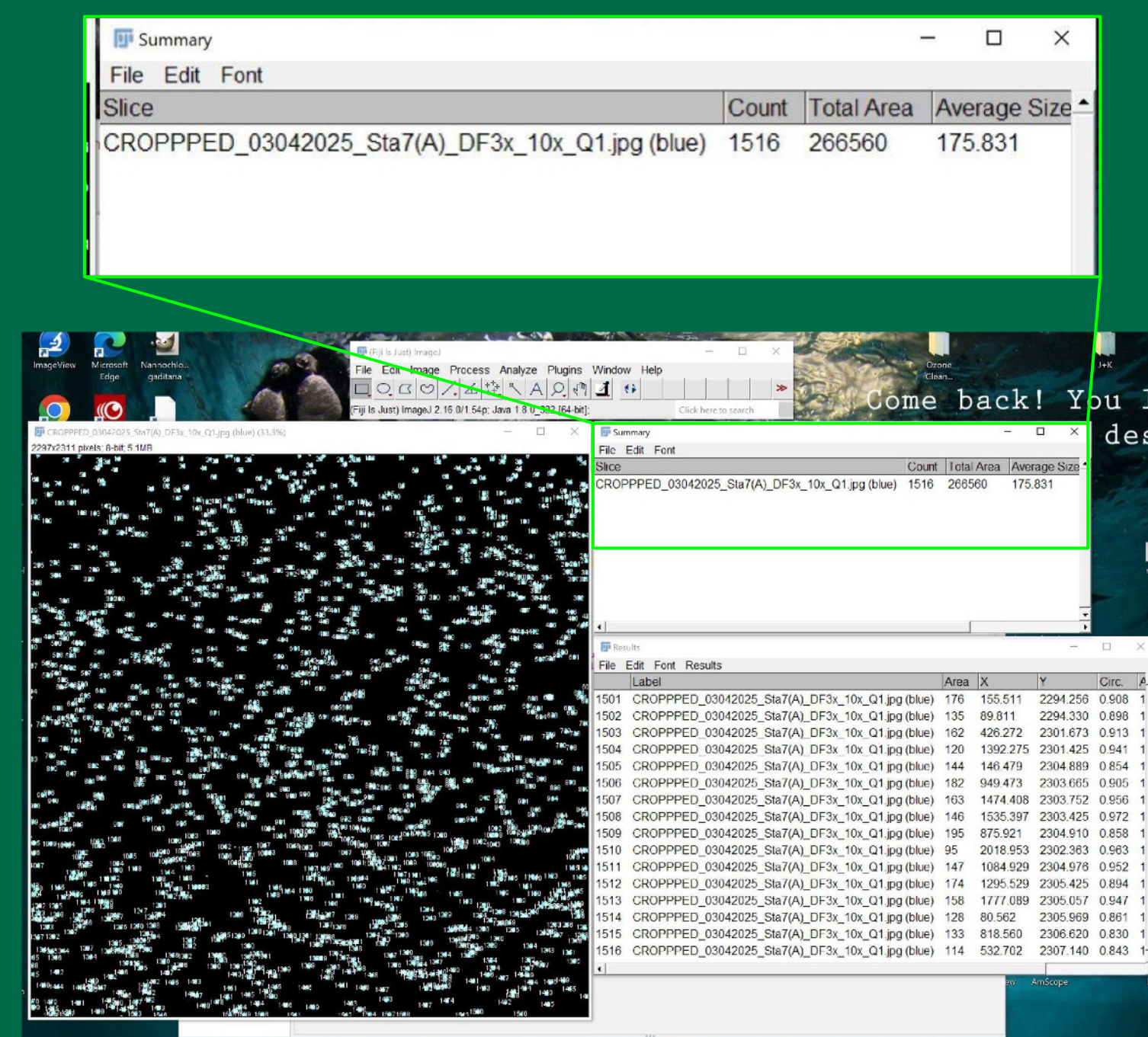

Step 1: Open Image

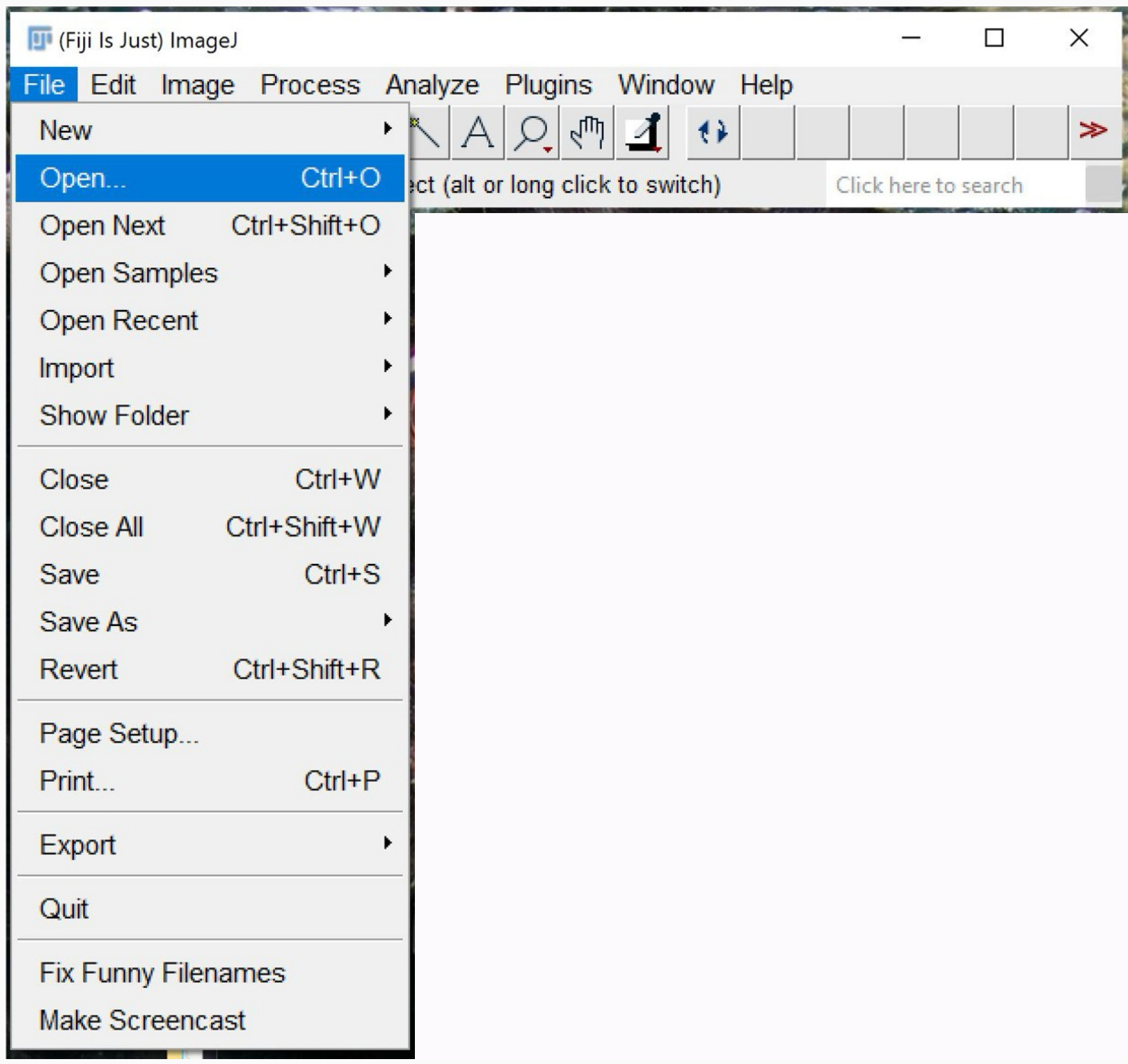

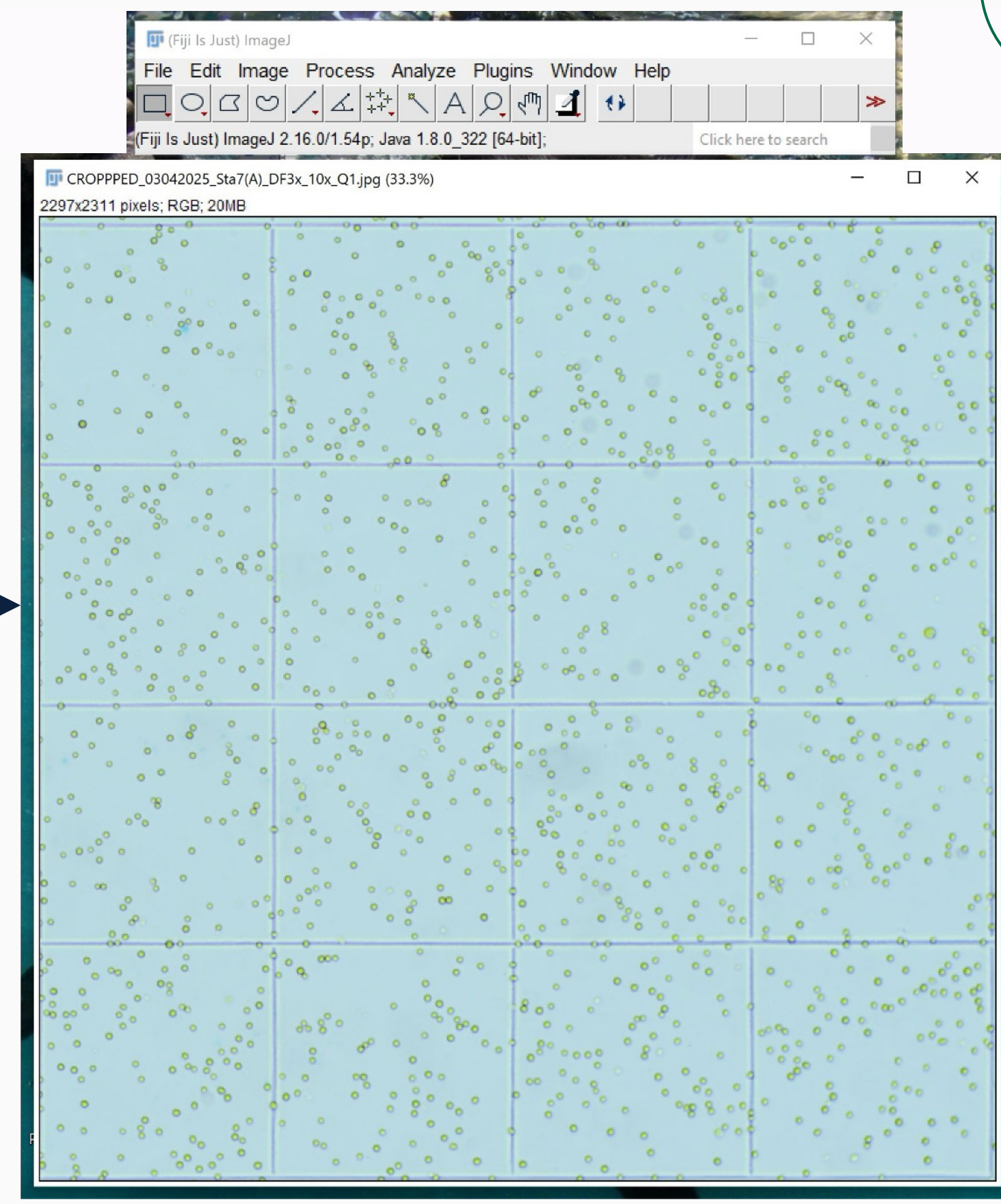

(Optional) Step 2: Adjust Brightness/Contrast

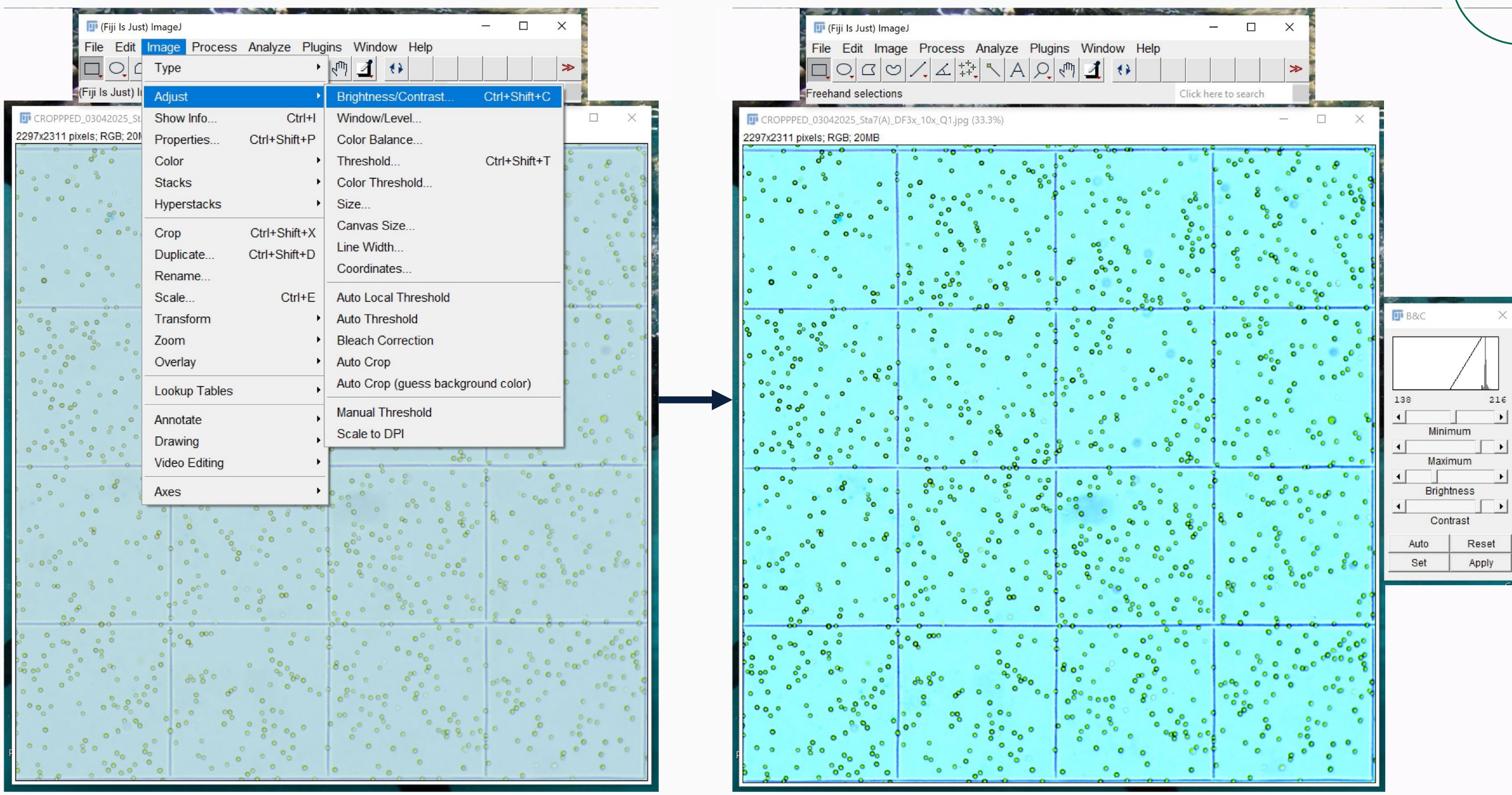

(Optional) Step 3: Split Channels

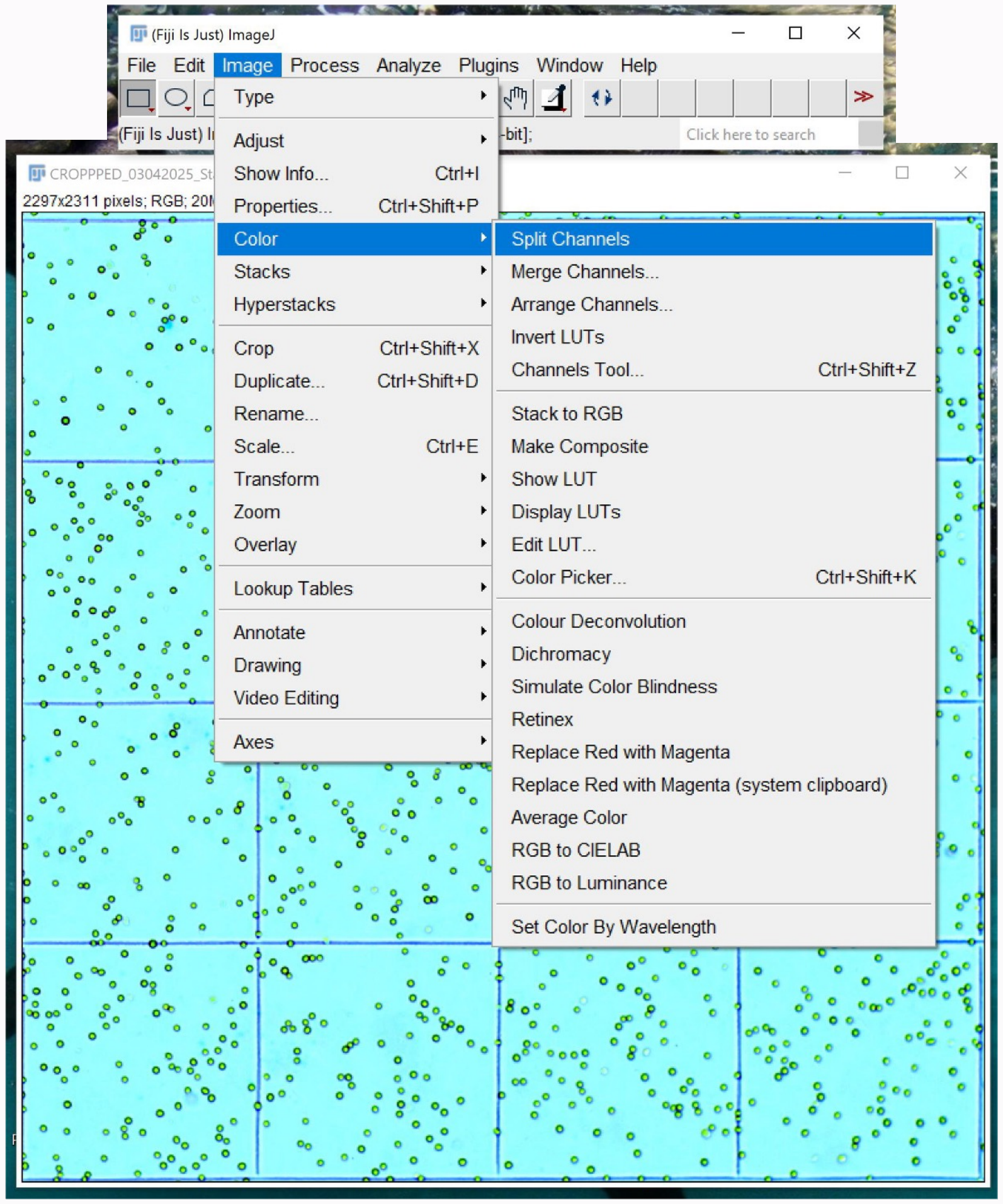

(Optional) Step 3: Split Channels

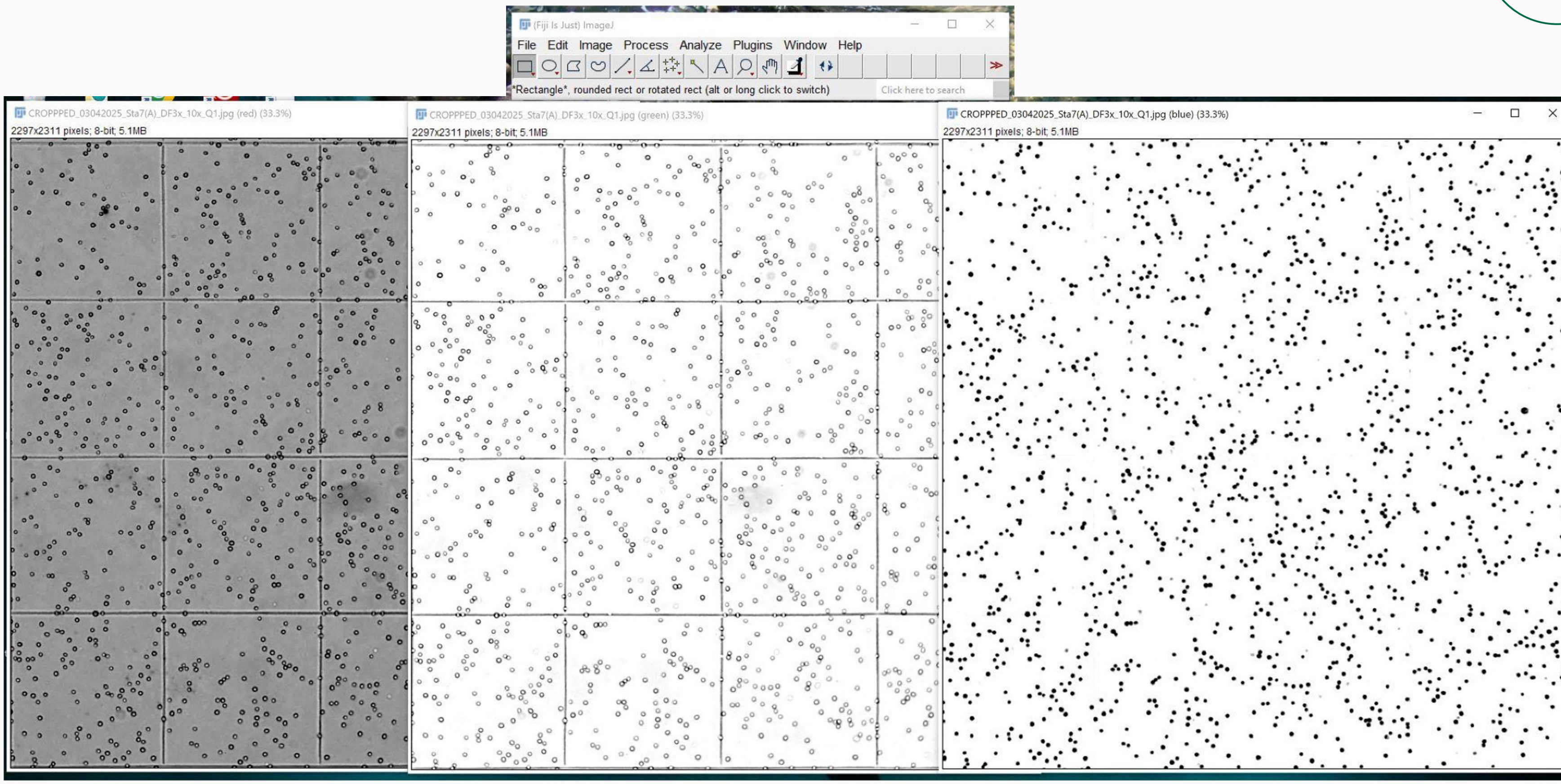

Step 4: Run Code

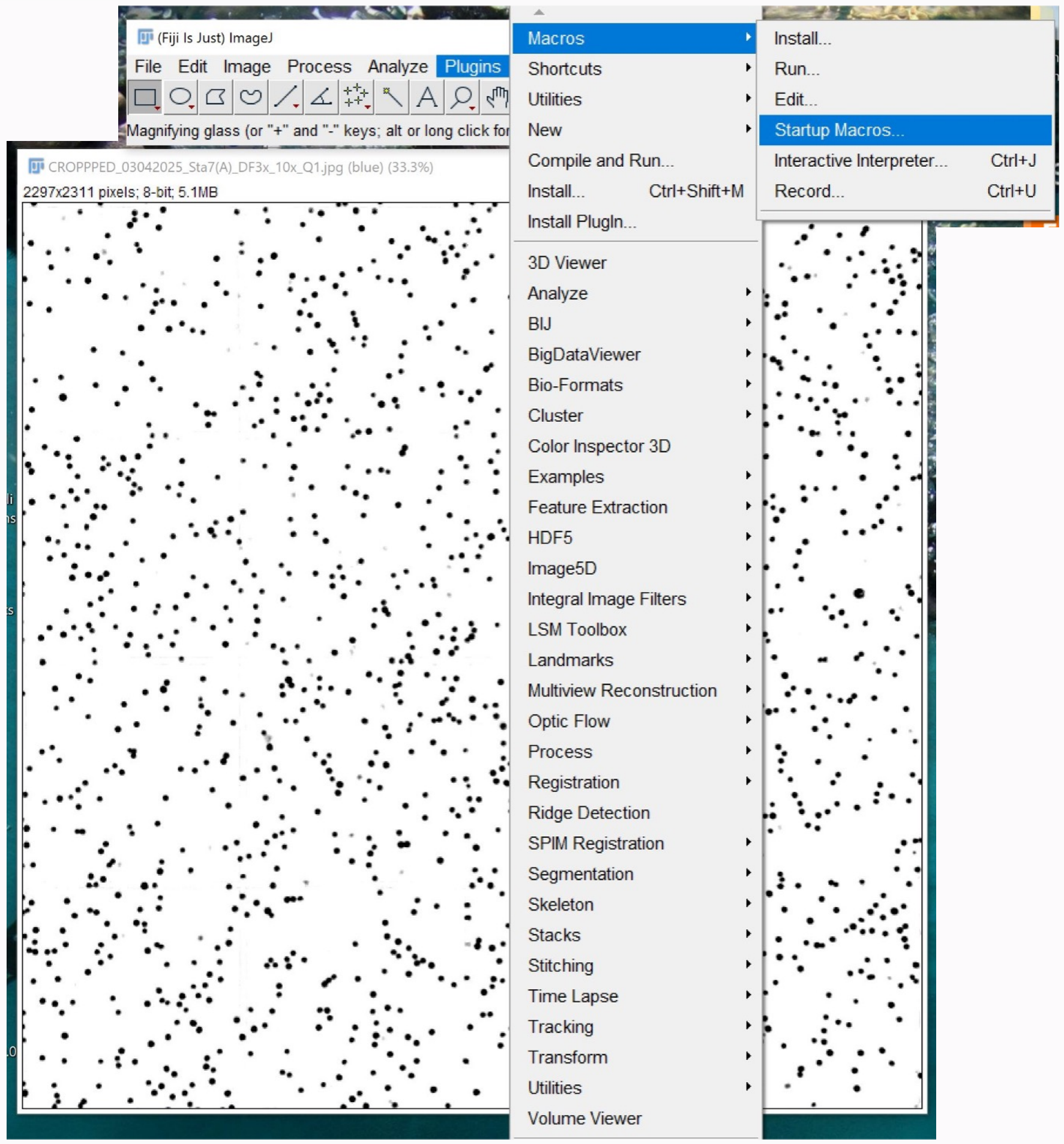

Step 4: Run Code

Threshold: Simple Segmentation

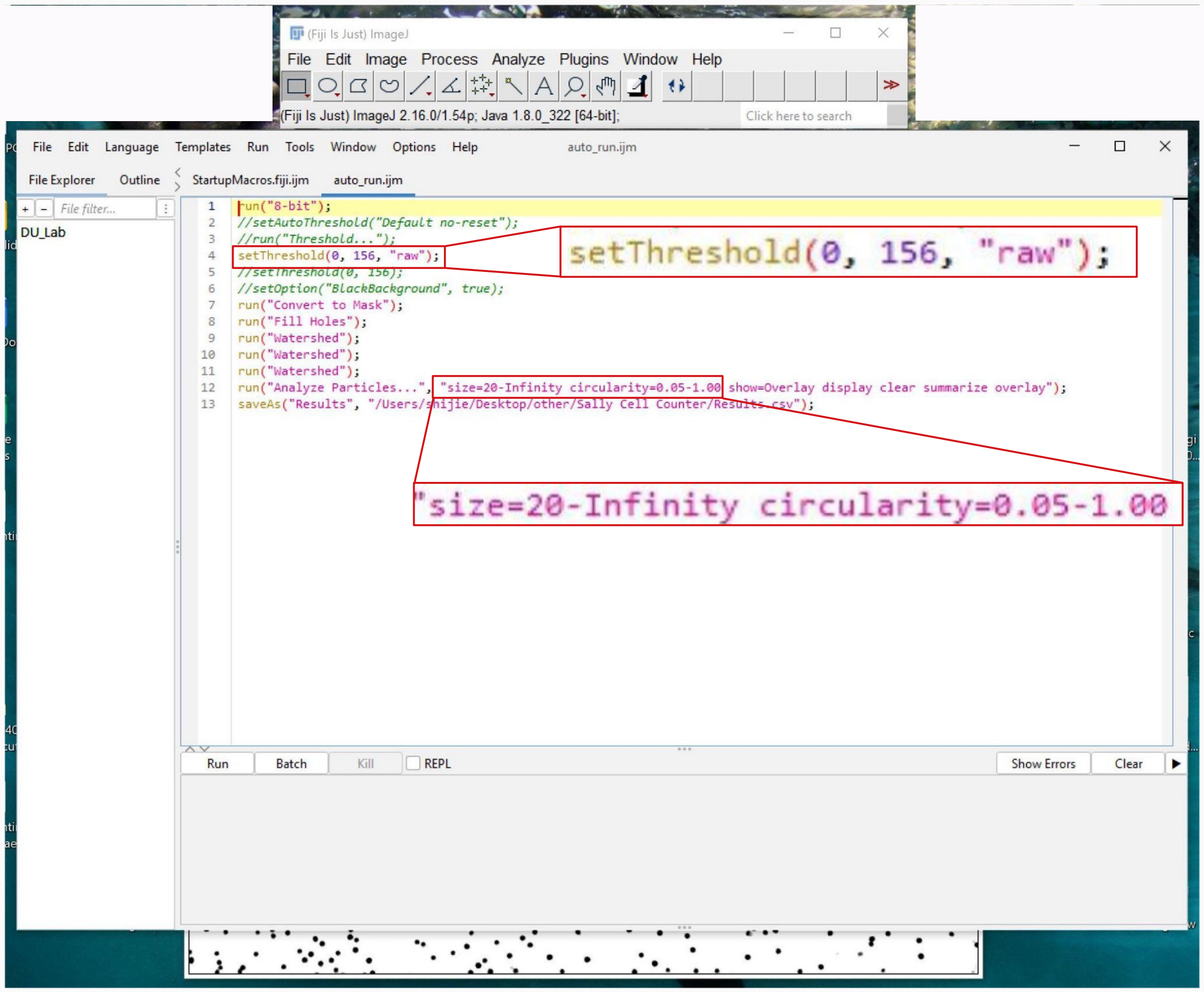

Size: How large are the pixels?

Circularity: How round are the pixels?

Record Results

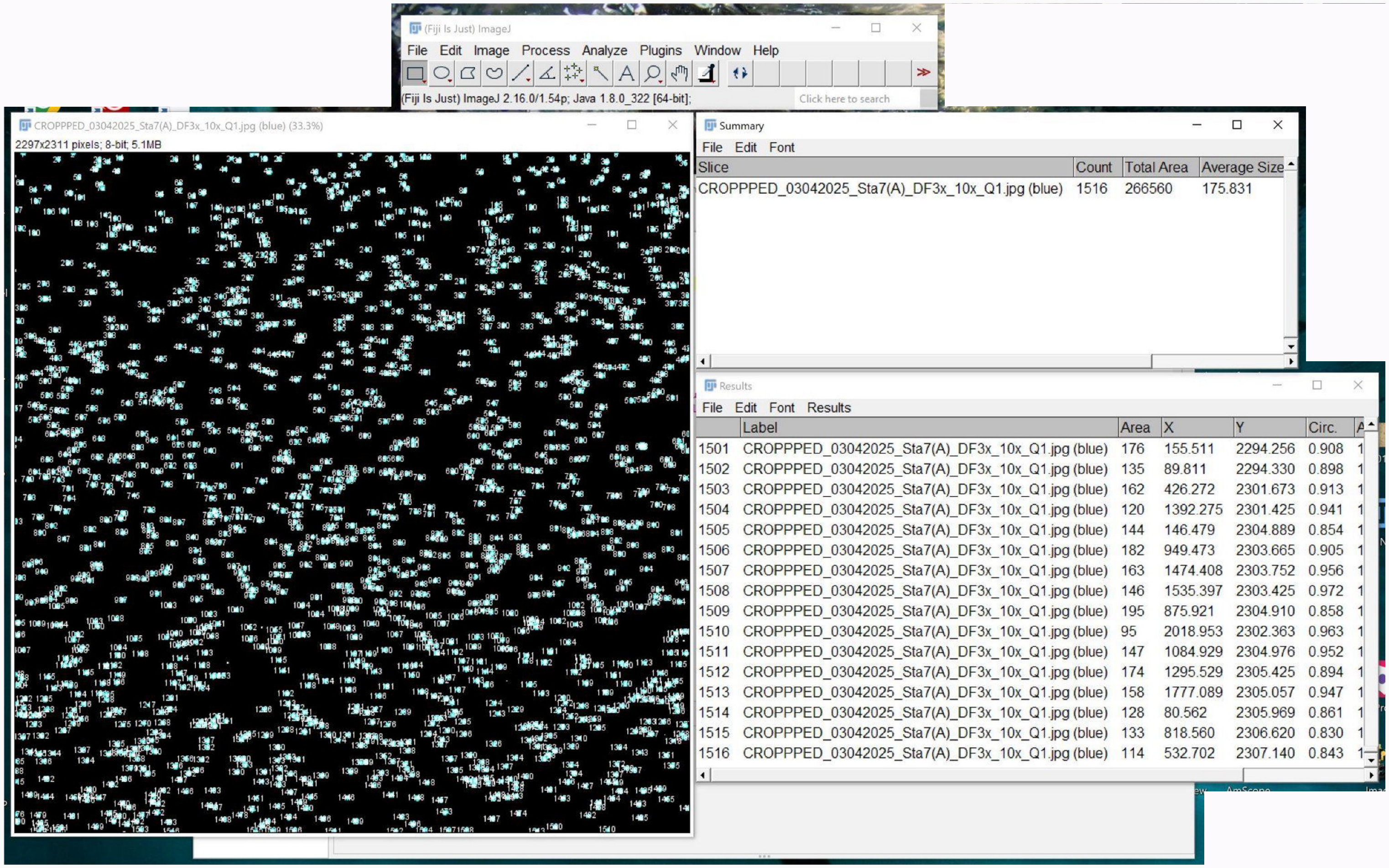

Slice	Count	Total Area	Average Size
CROPPPED_03042025_Sta7(A)_DF3x_10x_Q1.jpg (blue)	1516	266560	175.831

	Label	Area	X	Y	Circ.	A
1501	CROPPPED_03042025_Sta7(A)_DF3x_10x_Q1.jpg (blue)	176	155.511	2294.256	0.908	1
1502	CROPPPED_03042025_Sta7(A)_DF3x_10x_Q1.jpg (blue)	135	89.811	2294.330	0.898	1
1503	CROPPPED_03042025_Sta7(A)_DF3x_10x_Q1.jpg (blue)	162	426.272	2301.673	0.913	1
1504	CROPPPED_03042025_Sta7(A)_DF3x_10x_Q1.jpg (blue)	120	1392.275	2301.425	0.941	1
1505	CROPPPED_03042025_Sta7(A)_DF3x_10x_Q1.jpg (blue)	144	146.479	2304.889	0.854	1
1506	CROPPPED_03042025_Sta7(A)_DF3x_10x_Q1.jpg (blue)	182	949.473	2303.665	0.905	1
1507	CROPPPED_03042025_Sta7(A)_DF3x_10x_Q1.jpg (blue)	163	1474.408	2303.752	0.956	1
1508	CROPPPED_03042025_Sta7(A)_DF3x_10x_Q1.jpg (blue)	146	1535.397	2303.425	0.972	1
1509	CROPPPED_03042025_Sta7(A)_DF3x_10x_Q1.jpg (blue)	195	875.921	2304.910	0.858	1
1510	CROPPPED_03042025_Sta7(A)_DF3x_10x_Q1.jpg (blue)	95	2018.953	2302.363	0.963	1
1511	CROPPPED_03042025_Sta7(A)_DF3x_10x_Q1.jpg (blue)	147	1084.929	2304.976	0.952	1
1512	CROPPPED_03042025_Sta7(A)_DF3x_10x_Q1.jpg (blue)	174	1295.529	2305.425	0.894	1
1513	CROPPPED_03042025_Sta7(A)_DF3x_10x_Q1.jpg (blue)	158	1777.089	2305.057	0.947	1
1514	CROPPPED_03042025_Sta7(A)_DF3x_10x_Q1.jpg (blue)	128	80.562	2305.969	0.861	1
1515	CROPPPED_03042025_Sta7(A)_DF3x_10x_Q1.jpg (blue)	133	818.560	2306.620	0.830	1
1516	CROPPPED_03042025_Sta7(A)_DF3x_10x_Q1.jpg (blue)	114	532.702	2307.140	0.843	1
